# Relationship Between Nasal Trigeminal Receptor Expression and Trigeminal Sensitivity

**DOI:** 10.1002/oto2.70202

**Published:** 2026-02-11

**Authors:** Akshita Joshi, Yiling Mai, Susanne Füssel, Thomas Hummel

**Affiliations:** ^1^ Department of Otorhinolaryngology, Smell and Taste Clinic TU Dresden Dresden Germany; ^2^ Department of Urology TU Dresden Dresden Germany

**Keywords:** intranasal trigeminal function, lateralization task, nasal swab, trigeminal function measurement, trigeminal receptor, trigeminal sensitivity

## Abstract

An intact intranasal trigeminal function is crucial for chemosensory and somatosensory perception, environmental irritation detection, and triggering protective reflexes. Accurately assessing intranasal trigeminal function is thus essential. Since transient receptor potential (TRP) channels' activation mediates this function, their expression levels in the nasal mucosa may serve as potential indicators. The present study examined the relationship between intranasal trigeminal sensitivity, assessed via the lateralization task, and the messenger RNA (mRNA) expression levels of TRP channels using quantitative polymerase chain reaction (qPCR) in healthy individuals. The results indicated that individuals with lower lateralization scores exhibited significantly reduced TRPA1 mRNA expression levels, suggesting that TRPA1 density may influence behavioral responses to trigeminal stimulation. The findings provide promising evidence linking nasal TRPA1 expression to psychophysical measures, supporting the potential of nasal swabs as a simple, non‐invasive, biologically objective tool for assessing intranasal trigeminal function.

Trigeminal receptors, primarily found in the nasal and oral cavities, respond to chemical stimuli that elicit somatosensory experiences such as cooling, burning, or tingling. Many of them belong to the transient receptor potential (TRP) family, including TRPA1, TRPM8, and TRPV1.^1^ TRPA1 is activated by pungent compounds found in mustard, like allylisothiocyanate. TRPM8 responds to cooling agents like eucalyptol, while TRPV1 is sensitive to capsaicin, the burning component in chilis.^1^, ^2^ TRPV1 expressing neurons also co‐express TRPA1 and TRPM8.^3^

To quantify intranasal trigeminal sensitivity, trigeminal lateralization task (TLT) is used.^4^, ^5^ It assesses an individual's ability to localize stimuli presented unilaterally. Higher lateralization scores indicate greater trigeminal neural processing,^6^ suggesting a potential increased receptor engagement. This study investigates whether individuals with higher lateralization scores exhibit increased expression of TRP receptors.

## Methods

The study was approved by the Ethics Committee of the Medical Faculty at the Technical University of Dresden and conducted in accordance with the Declaration of Helsinki. Thirty‐six healthy adults (25.0 ± 2.8 years, 23 women) with normal olfaction were recruited and confirmed as normosmic using the “Sniffin' Sticks” identification test.^7^, ^8^ Nasal endoscopy was performed to ensure no structural abnormalities or infection. Participants then underwent TLT using a “squeezer” setup with two 250 mL polypropylene bottles, one containing eucalyptol (4 mL, 99%) and the other empty, each fitted with a soft silicone tube. During testing, participants held the tubing near the nasal valve region while the examiner squeezed the setup, delivering 15 mL of odor puff per nostril per trial simultaneously. After each trial, participants indicated the stimulated side. Twenty randomized trials (10 per side) were presented, and the final score was the total correct responses. Nasal swabs were collected for RNA extraction, taken from the antero‐medial surface of the middle turbinate, targeting the least obstructed and most accessible area within the nose. TRPA1, TRPV1, and TRPM8 messenger RNA (mRNA) expression levels were analyzed by quantitative polymerase chain reaction (qPCR) and normalized to the reference genes peptidylprolyl isomerase A (PPIA) and TATA‐box binding protein (TBP) using the 2−ΔΔCT method.^9^ The resulting normalized expression ratios (eg, TRPA1/TBP and TRPA1/PPIA) were used for statistical analysis, with higher ratios indicating greater receptor mRNA expression. Descriptive statistics, independent sample *t* tests, and Pearson correlation were used for statistical analyses.

## Results

**Figure 1:**
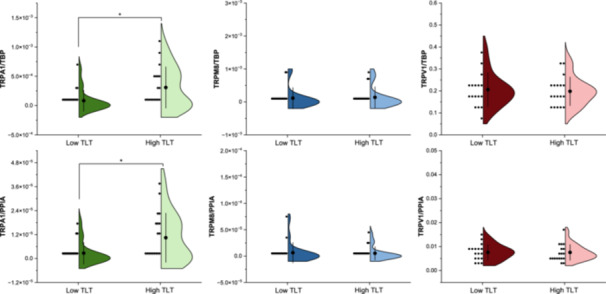
Transient receptor potential (TRP) expression levels in low‐ and high‐trigeminal lateralization task (TLT) groups. *Note*: Violin plots show normalized TRP messenger RNA (mRNA) expression. Dots: individual data points; black markers: mean; error bars: standard deviation; *P < .05.

We observed significantly different TRPA1 expression between high‐ and low‐TLT groups, divided based on the binomial above‐chance cutoff of 15 for the 20‐trial TLT. The low‐TLT group (TLT < 15, n = 17) exhibited lower TRPA1/TBP (8.39 × 10^−^^5^ ± 1.79 × 10^−^^4^ vs 3.08 × 10^−4^ ± 3.53 × 10^−^^4^; *t* = 2.39, *P* = .024, Hedges' *g* = 0.78) and TRPA1/PPIA (2.68 × 10^−^^6^± 5.49 × 10^−^^6^ vs 1.04 × 10^−^^5^ ± 1.23 × 10^−^^5^; *t* = 2.42, *P* = .024, Hedges' *g* = 0.78) compared to the high TLT group (TLT ≥ 15, n = 18). See Table 1 and Figure 1. Correlations between TLT scores and TRPA1 expression were not significant, but were close to the significant margin (*r* = 0.31‐0.33, *P* = .055‐0.069 across reference genes). Regarding the TRPV1 and TRPM8 expression, no significant group differences (*t* = 0.03‐0.32, *P* = .75‐0.98) nor correlations (r = −0.12 to 0.11, *P* = .47‐0.95) were observed.

**Table 1:** Descriptive Statistics^a^

	Total sample	High‐TLT group	Low‐TLT group
Sample size	36	18	18
Age	25.03 ± 2.81	25.28 ± 3.39	24.78 ± 2.16
Sex	23F:13M	13F:5M	10F:8M
TLT	15.44 ± 3.10	18.11 ± 1.64	12.78 ± 1.43
OT	7.85 ± 1.43	7.89 ± 1.21	7.81 ± 1.66
OI	14.16 ± 1.36	14.22 ± 0.88	14.11 ± 1.75
TRPA1/TBP	1.99 × 10^−4^ ± 3.00 × 10^−4^	3.08 × 10^−4^ ± 3.53 × 10^−4^	8.39 × 10^−5^ ± 1.79 × 10^−4^
TRPA1/PPIA	6.65 × 10^−6^ ± 1.03 × 10^−5^	1.04 × 10^−5^ ± 1.23 × 10^−5^	2.68 × 10^−6^ ± 5.49 × 10^−^^6^
TRPM8/TBP	1.27 × 10^−4^ ± 3.17 × 10^−4^	1.38 × 10^−4^ ± 3.21 × 10^−4^	1.14 × 10^−4^ ± 3.22 × 10^−4^
TRPM8/PPIA	5.85 × 10^−6^ ± 1.59 × 10^−5^	5.32 × 10^−6^ ± 1.26 × 10^−5^	6.41 × 10^−6^ ± 1.92 × 10^−5^
TRPV1/TBP	2.02 × 10^−1^ ± 7.03 × 10^−2^	1.98 × 10^−1^ ± 6.49 × 10^−2^	2.06 × 10^−1^ ± 7.74 × 10^−2^
TRPV1/PPIA	7.56 × 10^−3^ ± 3.24 × 10^−3^	7.54 × 10^−3^ ± 3.28 × 10^−3^	7.58 × 10^−3^ ± 3.29 × 10^−3^

Abbreviations: F, female; M, male; OT, Sniffin' Sticks odor threshold test; OI, Sniffin' Sticks odor inentification test; TBP and PPIA, housekeeping genes used to normalize target gene expression; TLT, trigeminal lateralization task; TRP/TBP and TRP/PPIA, normalized expression ratios of target genes (TRPA1, TRPV1, and TRPM8) relative to reference genes, with higher ratios indicating higher receptor mRNA levels.

^a^ Mean and standard deviation are presented for the total sample and separately for the high‐ and low‐TLT groups, categorized by whether participants had a TLT score above or below the 20‐trial cutoff (≥15 for high, <15 for low). One outlier exceeding three standard deviations from the mean was excluded separately for TRPA1, TRPV1, and TRPM8 expression analyses.

## Discussion

Individuals in the low‐TLT group exhibited decreased TRPA1 expression levels than those in the high‐TLT group, suggesting a link between psychophysical performance and receptor density. Given that the TLT used eucalyptol that activates TRPM8, it was initially hypothesized that TRPM8 expression would differ between the two groups. However, the observed group differences occurred only in TRPA1 expression. One possible explanation is the widespread co‐activation of TRPA1. Most odors commonly co‐activate TRPA1 (but not TRPV1) alongside other TRP channels,^10^ including eucalyptol that has been shown to activate TRPM8.^11^ In addition, rodent studies suggest that TRPA1, alongside TRPM8, contributes to both innocuous and noxious cold sensations, potentially serving as a complementary or synergistic cold transduction system,^12^ highlighting TRPA1's broader role beyond its well‐established involvement in nociception. Thus, TRPA1 expression may associate with eucalyptol TLT performance either directly through sensory transduction or indirectly via interactions with other TRP channels. A possible explanation for the lack of a significant association between eucalyptol TLT and TRPM8 expression could be related to the sampling method. Nasal swabs are non‐invasive and collect samples from the superficial mucosa, whereas TRPM8 may be more densely localized in deeper mucosal layers.^13^ This is supported by findings from a biopsy study that collected thicker samples (than the nasal swab) from the nasal mucosa, and found correlations between TRPM8 expression and eucalyptol TLT.^14^ Regarding the insignificant correlation, limited variability among healthy individuals might partly explain such result. Moreover, behavioral performance may not scale linearly with TRP density, as it may also be influenced by factors beyond receptor expression, like cognition.

These results support that nasal swab‐based TRP profiling may serve as a simple, non‐invasive method to assess intranasal trigeminal function and potentially help differentiate sensory dysfunction from structural nasal complaints, and monitor sensory recovery following infections or surgery. One limitation is the small sample size, which may increase the influence of individual variability and limit generalizability. Future research should include patients with various sinonasal conditions, like nasal obstruction, to better validate nasal swab‐based TRP profiling as a functional biomarker.

## Conclusion

Despite the relatively small sample size and the potential limitations of not being able to collect TRPM8 through superficial nasal swab, this study provides promising evidence linking nasal TRPA1 expression to psychophysical measures, supporting the potential of nasal swabs as a simple, non‐invasive, biologically objective tool for assessing intranasal trigeminal function. To confirm the robustness and assess clinical relevance, future studies should include pathological cases, examine TRP density alongside other trigeminal measures, and employ larger samples.

## Author Contributions

**Akshita Joshi**, investigation, methodology, writing—original draft, writing—review & editing; **Yiling Mai**, data curation, formal analysis, methodology, visualization, writing—original draft, writing—review & editing; **Susanne Füssel**, methodology, writing—review & editing; **Thomas Hummel**, conceptualization, investigation, funding acquisition, project administration, supervision, writing—review & editing.

## Disclosures

### Competing interests

The authors declare no conflicts of interest.

### Funding source

The study received intramural funding through the Department of Otorhinolaryngology of the TU Dresden.
